# Supplementary material for: Orbitofrontal cortex computes gaze-dependent comparisons between attributes rather than integrated values
Source: PLoS Biol. 2025 Aug 7;23(8):e3003281. doi: 10.1371/journal.pbio.3003281 (PMC12331056; doi:10.1371/journal.pbio.3003281)
Supplement: Attachment — Submitted filename: REVIEWS-v2.pdf [file pbio.3003281.s010.pdf]

RESPONSE TO REVIEWS:

We thank the Reviewers for their time and thoughtful comments that have helped us revise our manuscript. Below, we respond to each comment in turn, and outline the changes we've made. Reviewer comments are in gray and new text highlighted in red in the excerpts and in the revised manuscript.

Reviewer #1: This paper reports data from monkeys making multi-attribute choices with neuronal recordings in OFC. The task involves sweetness and probability as independent dimensions. The key result is that (1) neurons encode the attended features not the integrated values, and (2) neurons encode the featural value difference. In addition, (3) the authors report clear attentional/ gaze effects. The authors conclude that OFC computes comparisons among attributes. The integration of dimensions into abstract value signals is a core issue - arguably THE core issue in neuroeconomics, and the OFC is a critical area for these questions. So the importance of the questions is above reproach. Overall, I was persuaded that the task (which is clever and powerful) is appropriate to the questions asked, and that the data are compelling and the analyses are done well.

In summary, this is an excellent paper that in my opinion could be published as it. Despite this, I nonetheless will provide some opinions, although I want to make clear these are suggestions rather than requirements for my approval, which I am already happy to give.

Thank you for the thoughtful assessment and positive comments.

Most importantly, I felt that the core message of the paper is a bit muddled. This may be deliberate, since the findings are likely to be controversial and the authors may be eager to avoid controversy to achieve publication. If that is the case, I respect the decision, but in my view the authors are hiding their candle under a bushel.

To be specific, the authors findings are potentially quite revolutionary with respect to the role of OFC in economic choice. There is, to be sure, a moderate literature on this topic, including some of the authors' own work, and the authors do a very good job of citing this and summarizing it in the Discussion. In my view the standout paper in that set is Hunt et al., 2014. Other relevant ones include Tianming Yang's 2018 paper, and Vince McGinty's two papers. Those are all cited here, but, I felt that, together, they along with this paper, paint a portrait of OFC that is at odds with the generally accepted view of value integration, and that could be woven more clearly into the Introduction and Discussion to make a stronger case for importance. In particular, I left wanting a clearer answer to the question of "If OFC doesn't compute and compare abstract values then what does it do instead?" beyond simply a summary of all the results.

It's a welcome critique that the importance of our work is undersold! Our view is in line with the Reviewer's interpretation - that the results are fundamentally at odds with the generally accepted idea that value integration in OFC is a key computation in choices. There were two main reasons that caused us to tread lightly. First, it may be that there is flexibility in choice mechanisms, and how the decision is presented and/or the options available could influence how a choice is computed. There could be other cases where value integration is performed. However, we wish to argue against the default assumption that choices depend on computing an integrated value, which is the approach in most studies recently, and instead recommend a more thorough and thoughtful approach to empirical data and models of choice. The second qualifier is that we do see evidence that OFC neurons encode an integrated value signal, but only later in the choice and only for the chosen outcome, which leads to the interpretation that this signal reflects the choice outcome rather than the process of arriving at a decision. This is difficult to prove definitively, but would be a consistent explanation for these results as well as many others in the literature. Again, moving away from the default assumption that all choices depend on value integration would help clarify the role of value signals in different data sets. We've made a number of revisions to more strongly contrast our results with the prevailing notion of value integration.

In addition, with the Reviewer's encouragement, we've speculated further in the Discussion on how one might think about OFC function if it's not computing and comparing abstract values. We think that a useful framework comes from theories of embodied decision-making (e.g., Cisek and others), in which processing is determined by ongoing interactions the individual has with their environment. In the case of value-based decision-making,

these interactions generally take the form of seeking out or attending to information that helps form the decision. The idea that OFC is participating in such a process would explain the attention-dependent nature of OFC responses that we and others report.

Since the edits are fairly extensive, rather than reproduce them here, we refer the Reviewer to the red text in the Introduction and Discussion sections.

I have sometimes wondered whether what appear to be value comparison signals are instead normalized value signals (normalized relative to the other values on the menu). Those would look nearly identical in the data, but would have a very different meaning - they JUST encode the attended value (albeit normalized) and have no direct role in comparison.

This is an interesting point, and is related to our thoughts in the Discussion (now third paragraph) where we discuss divisive normalization. The Reviewer is correct, that the inverse coding we found here would be predicted by a divisive normalization mechanism, although there are two features of the results that we think are still very interesting, even if we assume a normalization mechanism is at play. The first is that, if normalization is occurring, it specifically involves the attended attribute being normalized to the like attribute of the other option (e.g., attended sweetness normalized by unattended sweetness), which is not necessarily anticipated by general divisive normalization processes. In response to another question from Reviewer 2 below, we have confirmed that the same degree of inverse coding is not found between the attended attribute and unmatching attribute of the other option, which means that, if the results are evidence of a normalization mechanism, it has attribute specificity, rather than being a general normalization of value. The second interesting point is that divisive normalization has been suggested as a potential computation involved in value comparison. For example, Yamada et al. (*Nat Commun*, 2018) reported that firing patterns in medial OFC neurons are consistent with divisive normalization, but the normalization is much more prominent when the monkey makes a choice and less so when he's forced to choose one of the options. Together, we can't rule in or out a normalization process given the current results (the main alternative being some type of competitive inhibition). While it may be that the signal is normalized, this wouldn't exclude a role in comparison. We have added some comments to the discussion to further clarify these ideas:

> (*Discussion*)
>
> Divisive normalization of value representations has been described in the lateral intraparietal cortex for cues in versus out of a receptive field (52), as well as in medial OFC for risky and safe options (53), suggesting this is a potential mechanism in OFC as well. Interestingly, divisive normalization patterns in medial OFC were much weaker when the monkey was forced to choose one option, obviating option comparisons (53), suggesting that divisive normalization could be a mechanism underlying the comparison process.

SMALLER COMMENTS

In that vein, the authors' claim of possible multiple decision systems (L409) seems not really borne out by the data, since the authors seem to find contradictory, not complementary, results to these earlier studies. It seems the methods here are strong enough that the authors should have managed to replicate the earlier contradictory results, and their failure to do so raises the possibility the earlier ones were in error.

We think our original text in this section might have been unclear, or perhaps we haven't fully understood this comment. In the referenced passage we noted that previous studies had found inverse relationships among integrated values, but these are most consistently in other brain areas, including the ventromedial PFC and ventral striatum. We agree that if those coding schemes are used in OFC, we should have detected them in this data set, and went to great lengths to try to do so. Since they were not found in our data, the possibility we intended to raise is that OFC performs comparisons based on the focus of attention, and that in a multi-attribute choice, attention is directed to attributes separately. In contrast, another region such as vmPFC, may be specialized for comparing integrated values, with the dependance on attention as yet unclear. This was the intended context for the reference to multiple decision systems, however in the process of revising the

Discussion in response to the earlier comment, we have removed this particular passage. Inverse coding of integrated values in vmPFC is still discussed elsewhere.

"Neurons that encode the integrated value of a choice should have non-zero regression coefficients for both attributes of the chosen option, simultaneously with the same sign" Since you require two things to pass threshold, your effective alpha is 0.025, I think. So to get an effective alpha of 0.05, you would have to use a real criterion of 0.1. In other words, the criteria are too strict. (Not sure about this, it depends on how the analyses took place).

Yes, this is a fair point, and was also noted by Reviewer 2. To address this, we have changed the significance thresholds for each encoding pattern to equate the effective alphas. We chose an effective alpha of 0.01 for a single time bin, since the use of overlapping time bins would inflate this alpha a bit. Because there are different conjunctions of probabilities in the different encoding patterns that we assessed, the process of equating effective alphas was not entirely straight-forward, but the rationale is now thoroughly described in the Methods, and the results show that false discovery rates during the fixation period are now very similar across the different categories. Importantly, the overall pattern of results remains the same. If anything, the prevalence of neurons encoding two like attributes is a bit higher. When we revisited this analysis, we also decided to eliminate one of the encoding patterns that was almost entirely absent: inverse encoding of chosen and unchosen options. We reasoned that the lack of unchosen option encoding made it redundant to show that the unchosen option wasn't encoded with the chosen. Edited text and figures are as follows:

(*Methods*)

Significant encoding of a model term was defined as a non-zero beta coefficient. To compare encoding patterns consisting of conjunctions of attributes (e.g., both chosen attributes) to single attribute encoding, we defined significance thresholds based on a total false discovery rate of $p \leq 0.01$ in a given time bin, then required significance for at least 3 consecutive time bins. We selected this criterion because it produced false discovery rates during the fixation epoch of <5%. Single attribute encoding included any one of four model terms reaching threshold, so the significance criterion was $p \leq 0.0025$ for 3 time bins. For chosen option encoding, chosen sweetness and chosen probability must both be significant (likewise for unchosen option encoding), meaning the effective alpha is $alpha_{sweetness}*alpha_{probability}$. To achieve an effective alpha of 0.01, the threshold for each attribute was $p \leq 0.1$ for three time bins. For like attribute encoding (*i.e.*, both sweetnesses *or* both probabilities), the alphas of two conjunctive patterns are added, such that the effective alpha is $(alpha_{sweetnessA}*alpha_{sweetnessB}) + (alpha_{probabilityA}*alpha_{probabilityB})$. To achieve an effective alpha of 0.01, the significance criterion for each term was $p \leq 0.0705$ for three time bins. Equating the significance criteria for each model term, rather than the effective alphas, produced qualitatively similar results, except there were unequal false discovery rates prior to options onset.

(Results)

Neurons that encode the integrated value of a choice should have non-zero regression coefficients for both attributes of the chosen option, simultaneously with the same sign. Some neurons fit these criteria (**Fig 2a**), but this encoding pattern was relatively infrequent and accounted for approximately 10% of neurons at any time (**Fig 2b, S2e**). If the value of the chosen option was compared to the unchosen option, we would expect to see the two attributes of the unchosen option also jointly represented, but almost no neurons did this (**Fig 2b, S2e**). Instead, many neurons encoded the value of only one attribute, most frequently an attribute of the chosen option (**Fig 2a**, **S2b**). Single attribute responses were found in 20-30% of neurons in any time bin during the choice epoch, making them the most common type of response (**Fig 2b, S2e**).

(*Fig 2b*) (*Fig S2e*)

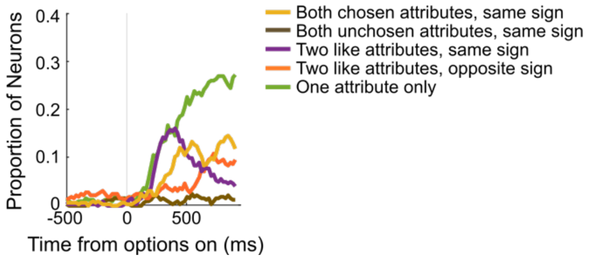

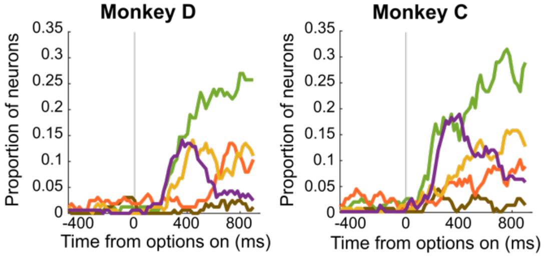

"Instead, many neurons encoded the value of only one attribute, most frequently an attribute of the chosen option" but this involves drawing an inference from a failure to achieve significance, which is not really valid with null hypothesis statistical testing, right?

Yes, it's true this statement is based on absence of evidence. To make a stronger case, we tested whether the 'One attribute only' neurons as a group are systematically influenced by the value of the paired attribute at levels that did not reach statistical significance. If there are sub-criterion effects consistent with value integration, we would expect a positive correlation between the putatively encoded attribute and other attribute. We found that only the neurons categorized as encoding 'Both chosen attributes, same sign' have a positive correlation between sweetness and probability. These figures are now included in a new panel of Figure S2.

(*Results*)

…many neurons encoded the value of only one attribute, most frequently an attribute of the chosen option (**Fig 2a**, **S2b**). These neurons showed no tendency for firing rates to be modulated by the value of the paired attribute in the same option (**Fig S2f**).

(*Figure S2f*)

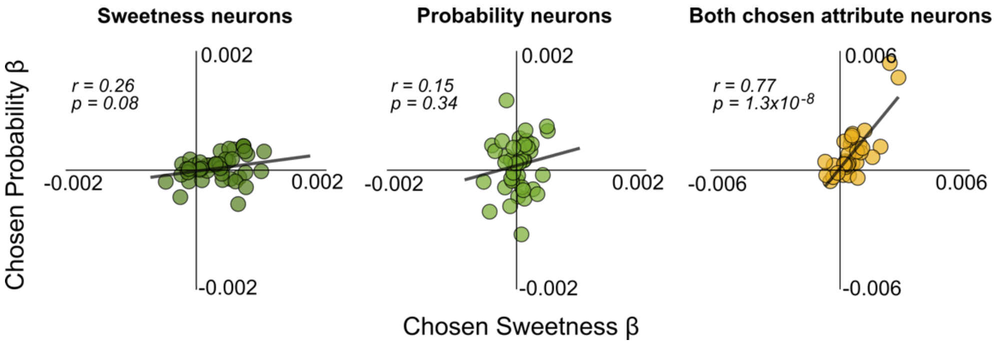

(**f**) Beta coefficients from the sliding regressions in (**e**), averaged across time windows with a leading edge 100 - 400 ms following the appearance of choice options. Neurons significantly modulated by chosen sweetness (dark green), chosen probability (light green) or both (yellow) in any of the included time bins are shown separately. Neurons considered selective for single attributes by these criteria (greens) had no overall tendency for firing rates to be modulated by the other attribute.

Finally, as a reviewer it would be easier to review with citations as names, not numbers.

This is understandable, but we have formatted the references following the style standard for the journal. Apologies!

Reviewer #2: This paper examines the coding and mechanisms of value comparison in orbitofrontal cortex

(OFC) using single unit recording in macaque monkeys, and in particular: (i) whether value coding is dependent upon integrated value comparison, as is often assumed/argued, or whether it may instead depend upon local comparisons within-attribute; (ii) how these comparisons and coding mechanisms are modulated by gaze. To achieve this, two macaque monkeys perform a task in which they compare two options of differing sweetness/probability, indicated by bar heights. A clever manipulation of bar height to attribute value means that the authors can find behavioural evidence of the choices being consistent with a within-attribute comparison strategy, rather than (or, perhaps, in addition to) an integrated-value comparison strategy. Perhaps more importantly, the paper finds compelling evidence that, at least in the context of the present task, gaze-dependent value comparison occurs within attribute within the OFC (figure 5c and 6b).

The findings from this paper are generally very robust and have important implications for working models of how we think about gaze-dependent value coding in the OFC. They offer a significant advance on previous studies that have attempted to address related questions. There are, however, a few points that I think the authors may wish to address before publication.

1. The most striking finding - the one that really convinced me of the authors' central claim - is shown in figure 5c. There is clear evidence for anticorrelation in beta coefficients between similar attributes across the two options, but not in the other comparisons that are tested (e.g. fixated/paired attribute, which might be positive if integrated values were being encoded). There were, however, a couple of frames of reference that I was surprised the authors didn't consider in the paper, and I think would be worth testing, to really examine whether there is *any* encoding of integrated values (either at the single neuron or population level).

a. The first of these is looking at the relationship between sweetness encoding and probability encoding. It is possible that when examining an option, there is a population code for the two attributes specifically, but this is not in the better/worse frame of reference for the two attributes (cf. figure 1g), nor in the fixated/paired frame of reference for the attributes (cf. figure 5c left panel), but instead is consistently in the sweetness/probability frame of reference. (In other words, when fixating an option, the same neurons will code for sweetness no matter whether it is the fixated attribute or the paired attribute). Can the authors try an analysis equivalent to figure 5c, with fixated sweetness on the x-axis and fixated probability on the y-axis? If there is no correlation between these two variables, it might provide further evidence against the notion of integrated value coding in this task.

This is an interesting idea. Our original model accounted for the identity of the attribute that the monkey fixated by including it as a binary regressor separate from the attribute value, but the question here is slightly different – whether neurons encode the sweetness or probability of an option that is fixated, regardless of which attribute in the option the monkey is gazing at. In order to address the reviewer's comment, we fit a different model where the regressors were the sweetness in the fixated option, probability in the fixated option, their respective mappings, and the same for the unfixated option. Given this model, we asked whether it was a better fit to neural activity than our original model. Since both had the same number of parameters, we could simply compare R^2 of the two. We found that nearly all neurons (84.7% and 82.4% for each monkey) were better fit by the original model, which included the attribute values that were fixated/paired/etc. regardless of whether the attribute identity was sweetness or probability. We think the new analysis is an excellent confirmation that the model we used is well-suited to the data, and we now include the results as a supplemental figure in the revised manuscript.

Given the results, however, we did not perform the comparison of sweetness and probability in the fixated option, since this appears to be the incorrect reference frame. That is, if neurons encode the fixated attribute regardless of its identity, and sometimes the fixated attribute is sweetness and sometimes it's probability, then the model that doesn't take into account which is the fixated *attribute* (but only the fixated option) will spread the fixated attribute's variance across the two regressors associated with the option and they will appear to vary similarly. This is illustrated in the toy example below, where blue/orange shading represents the attribute encoded positively/negatively.

Option A

Fixated	Paired
Swt 3	Prob 3
Swt 2	Prob 4
Prob 5	Swt 1
Swt 1	Prob 2

Option B

Like Att	Other Att
Swt 2	Prob 1
Swt 4	Prob 3
Prob 3	Swt 5
Swt 1	Prob 5

→

Fixated Option

Swt	Prob
Swt 3	Prob 3
Swt 2	Prob 4
Swt 1	Prob 5
Swt 1	Prob 2

Unfixated Option

Swt	Prob
Swt 2	Prob 1
Swt 4	Prob 3
Swt 5	Prob 3
Swt 1	Prob 5

Prob 4	Swt 4	Prob 2	Swt 2	Swt 4	Prob 4	Swt 2	Prob 2
Prob 3	Swt 5	Prob 1	Swt 2	Swt 5	Prob 3	Swt 2	Prob 1
Swt 2	Prob 3	Swt 5	Prob 1	Swt 2	Prob 3	Swt 5	Prob 1
Prob 2	Swt 5	Prob 2	Swt 1	Swt 5	Prob 2	Swt 1	Prob 2
Prob 1	Swt 2	Prob 3	Swt 4	Swt 2	Prob 1	Swt 4	Prob 3
…	…	…	…	…	…	…	…

The revised text and figures are shown below:

(*Results*)

We considered an alternative model, which hypothesizes that neurons are selective for the sweetness or probability in a gazed-at option, but insensitive to which attribute of the option the fixation is directed at. However, we found that this model fit the vast majority of neurons worse than the present one, indicating that OFC neurons preferentially encode attributes relative to gaze, regardless of the identity of the attribute (**Fig S3**).

(*Figure S3*)

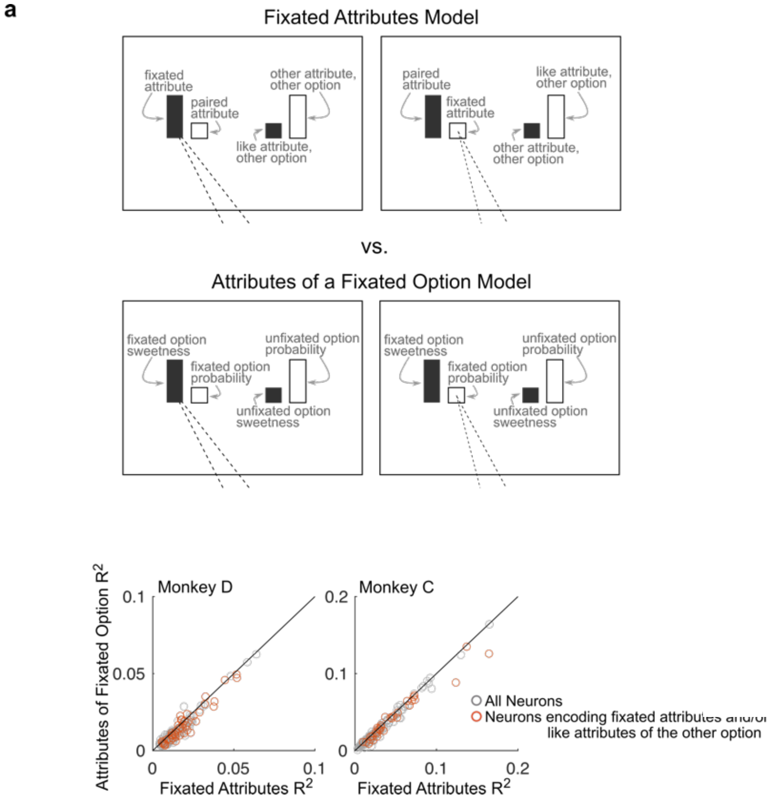

Supplemental Figure S3. OFC encodes attended attributes, not attributes of an attended option. We tested the possibility that OFC neurons selectively encode sweetness or probability, modulated by attention to an option. (**a**) Schematics of the two models that were contrasted, showing two fixation events. The model on the top assumes that neurons encode the value of any attribute in the focus of gaze and all other attributes on the screen are encoded in relation to that attribute. The model on the bottom assumes that neurons consistently encode either the sweetness or probability of the option that is fixated, regardless of which attribute of that option gaze falls on. (**b**) Scatterplots of whole model R^2's show that 84.7% (Monkey D) and 82.4% (Monkey C) of neurons are better fit by the Fixated Attributes Model. Red circles = neurons that met criteria for encoding either the fixated attribute or the like attribute of the other option in the main paper. Gray = all other neurons.

(*Methods*)

To test the alternative possibility that single neurons were selective for the value of sweetness or probability in the option that was fixated regardless of which attribute in that option the monkey directed its eyes to, we fit neurons to a second model (*Eq. 11*). In this model, FixOptSwt and FixOptProb are the magnitude of the sweetness and probability bar respectively

in the fixated option; UnfixOptSwt and UnfixOptProb are the sweetness and probability magnitudes in the unfixated option; FixOptSwtDir, FixOptProbDir, UnfixOptSwtDir, and UnfixOptProbDir are the mappings of each of these attributes using the same convention. The remaining regressors are as in *Eq. 10*.

(11) $$FRresid = \beta_0 + \beta_{FixOptSwt}(FixOptSwt) + \beta_{FixOptProb}(FixOptProb) + \beta_{UnfixOptSwt}(UnfixOptSwt) + \beta_{UnfixOptProb}(UnfixOptProb) + \beta_{FixOptSwtDir}(FixOptSwtDir) + \beta_{FixOptProbDir}(FixOptProbDir) + \beta_{UnfixOptSwtDir}(UnfixOptSwtDir) + \beta_{UnfixOptProbDir}(UnfixOptProbDir) + \beta_{FixAtt}(FixAtt) + \beta_{XPosFix}(XPosFix) + \beta_{YPosFix}(YPosFix)$$

Since *Eqs. 10* and *11* have the same number of parameters, we compared them by computing the whole model R^2 for each model fit to each neuron, averaging the sliding analysis in the same time window as shown in Fig 5c. Since nearly all neurons had higher R^2 for *Eq. 10*, we did not consider *Eq. 11* further.

b. Another possibility - perhaps less likely, but worth testing nonetheless - is that the currently fixated attribute is compared against *both* attributes of the other option at the same time. This would be a similar analysis to figure 5c, but with fixated attribute beta on the x-axis and (other attribute, other option) on the y-axis. In other words, for the middle two panels of figure 5c, what happens if we swap y-axes across these two panels - do the negative correlations now disappear?

This is also an interesting thought, and produced a weak negative correlation (r = -0.34), shown below. For comparison, the significant correlations reported in the manuscript (Fixated Attribute vs. Like Att, Other Option and Paired Attribute vs. Other Att, Other Option) have r-values of -0.83 and -0.61. This correlation, although present, had a significance test that barely survived multiple comparisons correction (the p-value in the figure is corrected; gray and black circles are the two different monkeys). Our interpretation, as the Reviewer suggests, is that there may be a small degree of comparison between the fixated attribute's value and the value of the unmatching attribute in the other option, but it is less prominent than the comparison with the matching attribute. For instance, perhaps this only happens occasionally, which would produce a weak effect across trials. Given that our interpretation remains that the strongest comparisons are between like attributes, we have opted not to include this result in the manuscript. However, if the Reviewer thinks that it adds important information, we would be happy to include it.

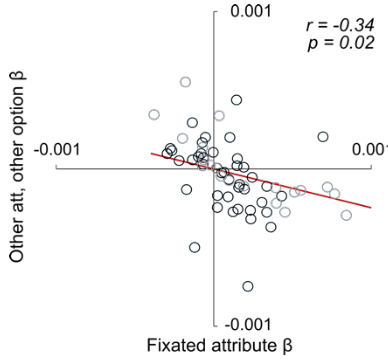

2. Although it is not central to the main claim of the paper, the test in figure 2b looks a little biased towards finding the result that is reported. It is easier to reject one null hypothesis (i.e. find 'one attribute only' value coding) than it is to simultaneously reject two null hypotheses (i.e. all the other possible coding schemes considered in this figure). This bias can actually be seen in the pre-stimulus baseline, where the proportion of neurons is green (false positives) is 0.05, as expected, whereas the proportion of neurons in the other colours is close to zero (as it is 0.05^2). The authors should think of a valid way to address this bias in the analysis.

Yes, this issue was also raised by Reviewer 1. To address it, we have changed the significance thresholds for each encoding pattern to equate the effective alphas. We chose an effective alpha of 0.01 for a single time bin, since the use of overlapping time bins would inflate this alpha a bit. Because there are different conjunctions of probabilities in the different encoding patterns that we assessed, the process of equating effective alphas was not entirely straight-forward, but the rationale is now thoroughly described in the Methods, and the results show that false discovery rates during the fixation period are now very similar across the different categories. Importantly, the overall pattern of results remains the same. If anything, the prevalence of neurons encoding two like attributes is a bit higher. When we revisited this analysis, we also decided to eliminate one of the encoding patterns that was almost entirely absent: inverse encoding of chosen and unchosen options. We reasoned that the lack of unchosen option encoding made it redundant to show that the unchosen option wasn't encoded with the chosen. Edited text and figures are as follows:

(*Methods*)

Significant encoding of a model term was defined as a non-zero beta coefficient. To compare encoding patterns consisting of conjunctions of attributes (e.g., both chosen attributes) to single attribute encoding, we defined significance thresholds based on a total false discovery rate of $p \leq 0.01$ in a given time bin, then required significance for at least 3 consecutive time bins. We selected this criterion because it produced false discovery rates during the fixation epoch of <5%. Single attribute encoding included any one of four model terms reaching threshold, so the significance criterion was $p \leq 0.0025$ for 3 time bins. For chosen option encoding, chosen sweetness and chosen probability must both be significant (likewise for unchosen option encoding), meaning the effective alpha is $alpha_{sweetness}*alpha_{probability}$. To achieve an effective alpha of 0.01, the threshold for each attribute was $p \leq 0.1$ for three time bins. For like attribute encoding (*i.e.*, both sweetnesses *or* both probabilities), the alphas of two conjunctive patterns are added, such that the effective alpha is $(alpha_{sweetnessA}*alpha_{sweetnessB}) + (alpha_{probabilityA}*alpha_{probabilityB})$. To achieve an effective alpha of 0.01, the significance criterion for each term was $p \leq 0.0705$ for three time bins. Equating the significance criteria for each model term, rather than the effective alphas, produced qualitatively similar results, except there were unequal false discovery rates prior to options onset.

(Results)

Neurons that encode the integrated value of a choice should have non-zero regression coefficients for both attributes of the chosen option, simultaneously with the same sign. Some neurons fit these criteria (**Fig 2a**), but this encoding pattern was relatively infrequent and accounted for approximately 10% of neurons at any time (**Fig 2b, S2e**). If this encoding of the chosen option was compared to the unchosen option, we would expect to see the two attributes of the unchosen option also jointly represented, but almost no neurons did this (**Fig 2b, S2e**). Instead, many neurons encoded the value of only one attribute, most frequently an attribute of the chosen option (**Fig 2a**, **S2b**). Single attribute responses were found in 20-30% of neurons in any time bin during the choice epoch, making them the most common type of response (**Fig 2b, S2e**).

(Fig 2b)

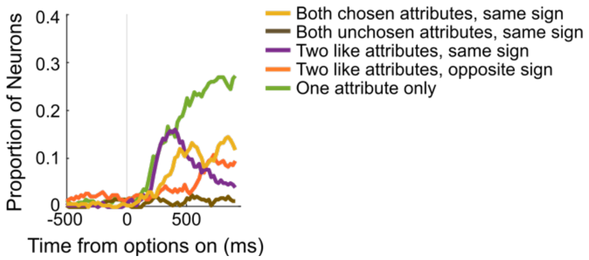

(Fig S2e)

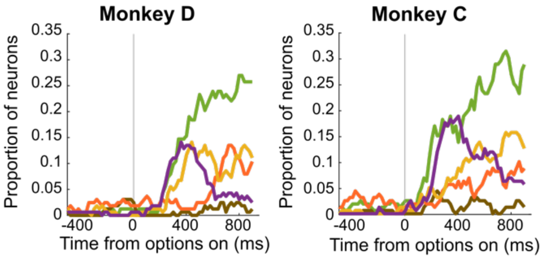

3. Figure 2f/g: here it might also be interesting to consider the sweetness/probability frame of reference mentioned above. I'm also unsure what time-window is used here - can the authors clarify this (and also make sure the timewindow is mentioned for all other correlations that aren't sliding analyses in the paper? Apologies if I missed this somewhere).

The time window includes 300 ms, starting 100 ms after the options appear – thank you for catching this omission. We have added the information in the Methods section:

To investigate relationships between beta coefficients for different encoded attributes, we averaged coefficients from the sliding regressions in time windows with a leading edge 100 to 400ms after the appearance of the choice options, and counted neurons with significance in any of the included bins as encoding neurons. This time range was chosen to encompass the majority of pre-choice data (the faster monkey had a median choice time of 520 ms), while allowing for visual processing delays after the options appeared and motor processing times before a choice was registered.

We have also included the comparison of sweetness and probability encoding among single neurons, similar to Figure 2f/g. Given the Reviewer's comments in point 1, we assume the goal they have in mind is testing whether the 'One attribute only' neurons are truly selective for the single attribute, or whether they are also influenced by the paired attribute, which would produce a positive correlation. Therefore, we tested these groups of neurons separately and found that only the neurons categorized as encoding 'Both chosen attributes, same sign' have a positive correlation between sweetness and probability. These figures are now included in a new panel of Figure S2.

(*Results*)

…many neurons encoded the value of only one attribute, most frequently an attribute of the chosen option (**Fig 2a**, **S2b**). These neurons showed no tendency for firing rates to be modulated by the value of the paired attribute in the same option (**Fig S2f**).

(*Figure S2f*)

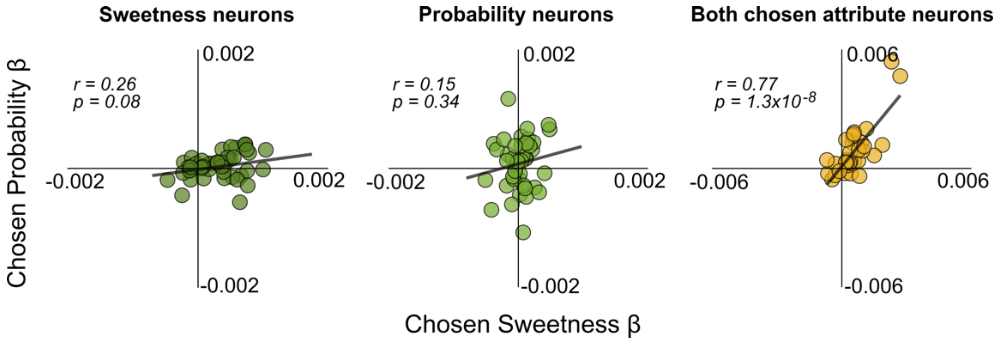

(**f**) Beta coefficients from the sliding regressions in (**e**), averaged across time windows with a leading edge 100 - 400 ms following the appearance of choice options. Neurons significantly modulated by chosen sweetness (dark green), chosen probability (light green) or both (yellow) in any of the included time bins are shown separately. Neurons considered selective for single attributes by these criteria (greens) had no overall tendency for firing rates to be modulated by the other attribute.

However, we note that we may have misinterpreted the Reviewer's question, as this isn't exactly a sweetness/probability reference frame – it's still the chosen/unchosen reference frame. (Or rather, the new figure panels are in the reference frame of sweetness/probability but the regression analysis that obtained the coefficients categorized the sweetnesses and probabilities according to whether they were chosen or unchosen). Given that there are always 2 sweetnesses and 2 probabilities, we're not clear how we could establish a pure sweetness/probability frame of reference. In other words, if we want to compare sweetness and probability, the question is which one of each is being compared? i.e., either chosen/unchosen; better/worse; fixated/unfixated; or location, which we didn't use because it has previously been shown to be a poor reference frame for OFC value encoding – e.g., see Rich & Wallis, 2016. Hopefully our new analysis answers the Reviewer's intended question, but we apologize if we missed it.

4. What is the distribution of eye-movement latencies? This seems potentially important for interpreting some of the results in figure 2, which are locked to option presentation rather than fixation.

The distribution of first and second fixation latencies relative to option presentation (so they can be compared to Fig 2) are shown in Figure S4a, which is reproduced below.

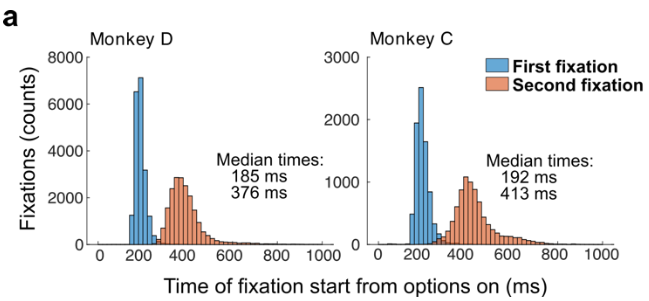

Reviewer #3: The fixation-triggered responses shown in Fig. 4 are very brief, and don't occur in many neurons. Likewise, the correlations in Fig. 5c are shown for only a small number of neurons. I would recommend showing and/or reporting in the main text the correlations in Fig. 5c when looking at all of the recorded neurons. If these effects are widespread, then at least the general trends should be evident at the population level (i.e. negative correlations in the blue and tan panels of Fig. 5c). If they are not widespread (i.e. if the population-level correlations are not significant) then this somewhat changes the overall interpretation of the results.

Fixation-triggered responses across all neurons, without thresholding for significance, are shown Figures 4d-e and Figure 6b and the associated supplemental Figures. These panels were included to show the generalizability of the reported effects across the entire population, as the Reviewer is asking about. Figure 6b is most directly related to Figure 5c, as it shows the strong inverse relationship between the fixated attribute and the like attribute of the other option in the entire population. We did not include the specific scatterplot panel that the reviewer requests because Figure 6b includes this information and more, as it shows the first and second fixations separately and across time. If we run the analysis in a static window as in 5c, the inverse relationship is extremely significant, in part because of the large number of data points (also discussed below).

I would also like to know whether the results in Fig. 4 and 5 differ for fixations onto sweetness vs. probability cues.

These effects hold if fixations on sweetness and probability are separated. Our original model accounted for the identity of the fixated attribute, so we anticipated that this was the case, but to confirm, we ran a new model that removed the identity regressor and separated fixations on sweetness and probability into two predictors. This partitioned the variance associated with the fixated attribute into two, but the inverse relationships between the fixated attribute and the like attribute of the other option were still clear (sweetness fixations: $r = -0.47$ $p = 3.8 \times 10^{-5}$. probability fixations: $r = -0.86$, $p = 1.0 \times 10^{-14}$).

As in the original analysis (related to the first part of the reviewer's question), the significance increased if the correlation included all neurons, rather than only those that met criteria for encoding of one of the two variables. At the same time, though, the correlation coefficient decreased, indicating (1) that the relationship holds at the population level, but (2) the extreme significance of the statistical tests is likely a result of the large sample size (sweetness fixations: $r = -0.36$ $p = 6.3 \times 10^{-11}$. probability fixations: $r = -0.64$, $p = 1.0 \times 10^{-38}$), which is why we chose to focus on neurons that met a criterion for encoding some relevant information. Also, the responses to a fixated sweetness and a fixated probability were positively correlated, as expected if neurons encode fixated values in general ($r = 0.35$, $p = 1.3 \times 10^{-10}$; all p-values uncorrected).
