# Supplementary material for: Orbitofrontal cortex computes gaze-dependent comparisons between attributes rather than integrated values
Source: PLoS Biol. 2025 Aug 7;23(8):e3003281. doi: 10.1371/journal.pbio.3003281 (PMC12331056; doi:10.1371/journal.pbio.3003281)
Supplement: Attachment — Submitted filename: Response to Reviews and Editorial Comments 062225.pdf [file pbio.3003281.s011.pdf]

## Response to Reviews and Editorial Comments

We thank the reviewers for the time they've taken to read our manuscript, and their supportive comments. Below are our responses to the remaining critiques. Reviewer/editor comments are in *gray* and new additions to the text are in red.

### R1

*I was positive about this paper before.*

*It is even stronger now.*

*I have no further concerns.*

### R2

*The authors have addressed all the comments I raised in the initial round of reviews. It is a great paper and makes a strong contribution. I'd be inclined for them to include the weak negative correlation in response to my previous point 1b, either in the main panels or in the supplement, but I'm happy for the authors to make the final call on this.*

We have added the figure from our previous RTR to the supplement as requested, and added a brief mention of this result to the main text. Given the request from R3 to present correlations of all neurons, in addition to the thresholded results in the main paper, we have also included an unthresholded scatterplot for this comparison.

(Results)

> In addition, there was a weaker tendency for fixated attributes to be negatively related to the other attribute of the other option, raising the possibility of a small degree of comparison between the fixated attribute's value and the value of the unmatching attribute in the other option (**Fig S6i-j**). In sum, our results demonstrate a consistent neural signature of value comparisons that are primarily among like attributes and depend on the monkeys' current focus of gaze.

(Supplemental Information)

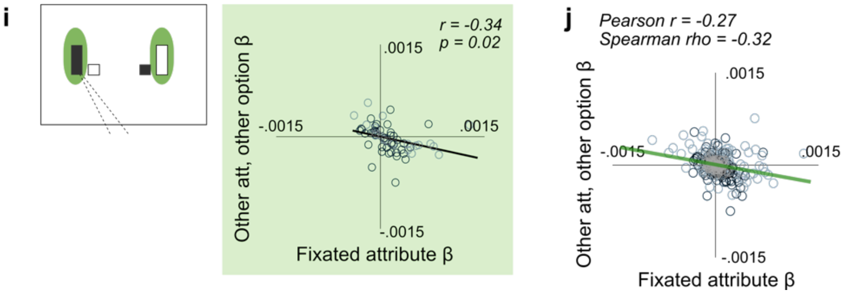


(**i**) Schematic and correlation for the comparison between the value of the fixated attribute and the non-matching attribute of the other option. Scatterplot includes only neurons significant for one or both of the compared attributes, as in the main paper. (**j**) The same scatterplot as (j), but with all recorded neurons. Black = Monkey D, Gray = Monkey C. Black = Monkey D, Gray = Monkey C. $p<10^{-5}$ for both correlation statistics.

### R3

*I thank the authors for answering my question in the response to reviews, but I would also like to see this*

*information reported in the manuscript. It's true that Figures 4d-e and 6b show that the mean activity is inversely related, but these graphs do not by themselves show the inverse relationship at the single cell level. The inverse relationship at the single-cell level is, in my opinion, the most interesting and important result in this paper, and it should be fully reported. The fact that the cell-level correlations are different for all neurons vs. just the neurons in 5c is an important finding that helps further the understanding of the relevant neural mechanisms.*

*To be explicit, I think the manuscript should show the correlations in Fig. 5c for all neurons, preferably in a main or supplemental figure; if the authors choose to report these in text rather than a supplemental figure, they should include both the Pearson's R as well as some outlier-resistance correlation measure, such as Spearman's Rho. I think the manuscript should also report the correlations in Fig. 5c (blue and tan panels) for sweetness and probability separately; it's fine if these statistics are just for the subset of neurons in Fig. 5c.*

We have included the requested scatterplots in new supplemental figure planes (Fig S6e-h), and report both Pearson's r and Spearman's rho statistics for each (the previous Fig S6e was moved to Fig S7 to accommodate the new panels). All results mirror the pattern shown in the main text figure 5, which slightly lower correlation statistics (due to adding neurons that are not significantly modulated) but more extreme p-values for the significant correlations (due to adding more degrees of freedom). As suggested, we also include the statistics for sweetness and probability separately in the main test, and added the model that produced them to the methods.

(Methods)

To test whether our main effects held when fixations on sweetness and probability were separated, we tested a model that removed the identity regressor and separated fixations on sweetness and probability into two predictors (*Eq. 11*). In this model, FixSwt and FixProb are the value of the fixated sweetness or probability, respectively, with 0 entered when a fixation was not directed at the relevant attribute.

$$(11) \quad FRresid = \beta_0 + \beta_{FixSwt}(FixSwt) + \beta_{FixProb}(FixProb) + \beta_{PairVal}(PairVal) + \beta_{LikeAttOtherOptVal}(LikeAttOtherOptVal) + \beta_{OtherAttOtherOptVal}(OtherAttOtherOptVal) + \beta_{FixDir}(FixDir) + \beta_{PairDir}(PairDir) + \beta_{LikeAttOtherOptDir}(LikeAttOtherOptDir) + \beta_{OtherAttOtherOptDir}(OtherAttOtherOptDir) + \beta_{FixAtt}(FixAtt) + \beta_{XPosFix}(XPosFix) + \beta_{YPosFix}(YPosFix)$$

(Results)

Consistent with comparisons of like attribute values, we found an inverse relationship between the beta coefficients of the fixated attribute and the like attribute of the other option (**Fig 5c**, blue). These effects held when fixations on sweetness and probability were separated in the model (sweetness fixations: $r = -0.47$, $p = 3.8\text{x}10^{-5}$, probability fixations: $r = -0.86$, $p = 1.0\text{x}10^{-14}$), and also when the comparison included all neurons without thresholding for significance (**Fig S6e-h**).

(Supplemental Information)

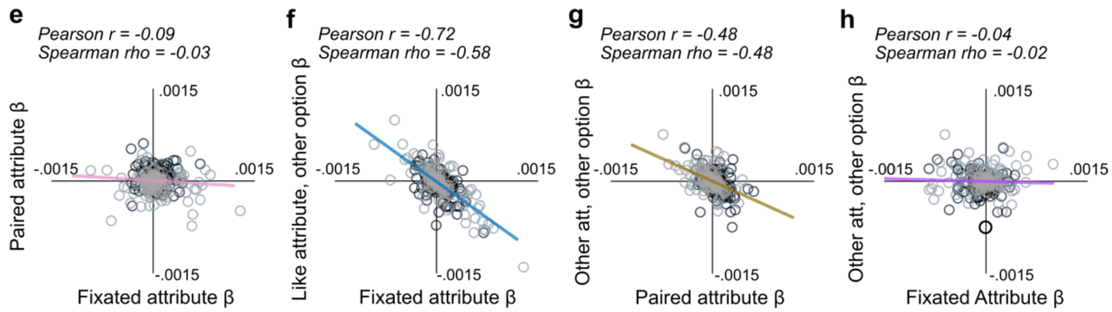

(**e-h**) Scatterplots of the comparisons shown in Fig 5c-f, including all neurons regardless of significance. Black = Monkey D, Gray = Monkey C. Pearson r and Spearman rho statistics are shown. P-values, Bonferroni corrected for 3 comparison each, are (**e**) both $p>0.3$ (**f**) both $p<10^{-10}$ (**g**) both $p<10^{-10}$ (**h**) $p>0.9$.

*A minor point, relevant to several figures: Figures with multiple sub-panels should have letter labels. For example, I would suggest that Fig. 5c use a separate letter for each of the four subpanels, i.e., 5c, 5d, 5e, 5f.*

We have renumbered the figure panels as requested.

**Response to editorial comments**

- We have updated our title for clarity and readability, following editorial consultation, as follows:

Orbitofrontal cortex computes gaze-dependent comparisons between attributes rather than integrated values

(Short Title: Gaze-dependent attribute comparison in orbitofrontal cortex)

----------------------------------------------------------------------

- *ETHICS STATEMENT:*

*-- Your article needs to include an Ethics Statement in the Methods section. Please add this (we see that you mention this in the "subjects" section of the methods) and ensure that it is consistent with the following guidelines:*
*-- Please include the full name of the IACUC/ethics committee that reviewed and approved the animal care and use protocol/permit/project license. Please also include an approval number.*
*-- Please include the specific national or international regulations/guidelines to which your animal care and use protocol adhered. Please note that institutional or accreditation organization guidelines (such as AAALAC) do not meet this requirement.*

The ethics statement has been amended to read as follows:

All procedures were in accord with the National Institutes of Health Office of Laboratory Animal Welfare (OLAW) guidelines and were approved by the Icahn School of Medicine at Mount Sinai Animal Care and Use Committee (Animal Welfare Assurance reference number D16-00069 (A3111-01)), under protocol #2017-0084.

----------------------------------------------------------------------

- *We see that you have included a Data and Code Availability statement, where you say that you will make these items available before publication. Please now make these items available so that we can examine them before your article is formally accepted. Please ensure that these items are consistent with the following guidelines:*

----------------------------------------------------------------------

- *CODE POLICY*

*Per journal policy, if you have generated any custom code during the course of this investigation, please make it available without restrictions. Please ensure that the code is sufficiently well*

*documented and reusable, and that your Data Statement in the Editorial Manager submission system accurately describes where your code can be found.*

All data and original code have been uploaded to the Dryad repository. A preview can be found at: http://datadryad.org/share/v-Bgo6UUaR3kOTYepp3Q2iL_n0OyDicU1hMqDU7Kz9E. The doi has been added to the manuscript:

Data and original analysis code are available at doi: 10.5061/dryad.f1vhhmh7t

--------------------------------------------------------------------

- *SPECIES INDICATED IN THE ABSTRACT?*
  *- We see that your abstract indicates that your study was done on monkeys. Can you please specify the species? Please note that per journal policy, the model system/species studied should be clearly stated in the abstract of your manuscript.*

  We have added the genus and species to the abstract.

  --------------------------------------------------------------------

- *As you address these items, please take this last chance to review your reference list to ensure that it is complete and correct. If you have cited papers that have been retracted, please include the rationale for doing so in the manuscript text, or remove these references and replace them with relevant current references. Any changes to the reference list should be mentioned in the cover letter that accompanies your revised manuscript.*

  We have double-checked the reference list.

Additional notes:

- We found and corrected a typo where a statistical result for one monkey was reported for the other.
- We adjusted the wording of the funding acknowledgement as requested.
- We have removed the main and supplemental information figures from the manuscript and uploaded them as separate files. The main and supplemental legends still appear in the main manuscript before and after the references, respectively.
